# Insights on the host associations and geographic distribution of *Hymenolepis folkertsi* (Cestoda: Hymenolepididae) among rodents across temperate latitudes of North America

**DOI:** 10.1007/s00436-016-5255-3

**Published:** 2016-09-14

**Authors:** E. P. Hoberg, A. A. Makarikov, V. V. Tkach, S. Meagher, T. N. Nims, R. P. Eckerlin, K. E. Galbreath

**Affiliations:** 1 Animal Parasitic Diseases Laboratory, Agricultural Research Service, USDA, Bldg 1180 BARC East, 10300, Baltimore Avenue, Beltsville, MD USA; 2 Institute of Systematics and Ecology of Animals, Siberian Branch Russian Academy of Sciences, Frunze Str. 11, Novosibirsk, 630091 Russia; 3 Department of Biology, University of North Dakota, 10 Cornell Street, 58202 Grand Forks, ND USA; 4 Department of Biological Sciences, Western Illinois University, 61455 Macomb, IL USA; 5 Science Department, Perimeter College at Georgia State University, 239 Cedar Lane, 30014 Covington, GA USA; 6 Mathematics, Science and Engineering Division, Northern Virginia Community College, 22003 Annandale, VA USA; 7 Department of Biology, Northern Michigan University, 1401 Presque Isle Ave., 49855 Marquette, MI USA

**Keywords:** *Hymenolepis folkertsi*, North America, Hymenolepidid tapeworms, Diversity, Faunal assembly

## Abstract

Synoptic data and an understanding of helminth parasite diversity among diverse rodent assemblages across temperate latitudes of North America remain remarkably incomplete. Renewed attention to comprehensive survey and inventory to establish the structure of biodiverse faunas is essential in providing indicators and proxies for identifying the outcomes of accelerating change linked to climate warming and anthropogenic forcing. Subsequent to the description of *Hymenolepis folkertsi* in the oldfield mouse, *Peromyscus polionotus*, additional specimens of hymenolepidids were collected or discovered in archived museum repositories from multiple species of deer mice (*Peromyscus maniculatus*, *Peromyscus leucopus*), the golden mouse (*Ochrotomys nuttalli*), chipmunks (*Tamias striatus*, *Tamias amoenus*), the 13-lined ground squirrel (*Ictidomys tridecemlineatus*), and tree squirrels (*Sciurus carolinensis*, *Sciurus niger*) from disjunct localities in the USA spanning southern Georgia, Virginia, Pennsylvania, Connecticut, the Upper Peninsula of Michigan, Wisconsin, and central Idaho. Specimens were largely consistent morphologically with the original description of *H. folkertsi*. Initial DNA sequence data, from a portion of the mitochondrial NADH dehydrogenase subunit 1, demonstrated intraspecific variation among three apparently geographically isolated populations attributed to *H. folkertsi* (uncorrected genetic distances of 2.7 % (Idaho and Michigan), 2.4 % (Virginia + Pennsylvania and Michigan), and 1.89 % (VA + PA and ID). Geography rather than host association explains the distribution and occurrence of *H. folkertsi*, and host colonization among deer mice, chipmunks, and other sciurids within regional sites is indicated. Genetic divergence revealed across localities for *H. folkertsi* suggests historically isolated populations, consistent with extended evolutionary and biogeographic trajectories among hymenolepidids and species of *Peromyscus* and *Tamias* in North America. Field inventory, that revealed these parasite populations, substantially alters our understanding of the distribution of diversity and provides insights about the nature of the complex relationships that serve to determine cestode faunas in rodents.

The diversity, structure, and assembly of helminth faunas characteristic of a temporally and spatially complex assemblage of rodent hosts across North America and the broader Holarctic region has been receiving increasing attention over the past decade (e.g., Cook et al. 2005; Makarikov et al. 2012, 2013; Hoberg et al. 2012; Haukisalmi et al. 2014; Gardner et al. 2014). Hymenolepidid tapeworms are recognized components of faunas associated with Cricetidae, Muridae, Geomyidae, Dipodidae, and Sciuridae, and species attributable to *Hymenolepis* Weinland, 1858 and *Arostrilepis* Mas-Coma and Tenora, 1997 remain to be fully evaluated, suggesting a need for broadened survey and inventory to document patterns of diversity especially at boreal to temperate latitudes of North America (e.g., Brooks et al. 2014).

An increasingly comprehensive understanding of diversity among rodent helminth faunas remains confounded by poor availability of voucher specimens linked to a diverse series of surveys over the past century (Makarikov et al. 2015). For example, considering the species-rich genus *Peromyscus* Gloger, (Cricetidae, Neotominae), representative series of voucher specimens of hymenolepidid cestodes and other helminths were only sporadically deposited following many local to regional surveys across North America (e.g., Erickson 1938; Hansen 1950; Grundmann and Frandsen 1960; Babero and Matthias 1967; Vaughn 2013). Further, many studies provided only incomplete identification to the generic level, leaving substantial gaps in our documentation of the fauna. Consequently, new field collections that provide access to comparative materials for integrated morphological/molecular analyses are essential to enhance our ability to more completely document parasite faunal diversity among Nearctic rodents (e.g., Haukisalmi et al. 2010; Hoberg et al. 2012; Gardner et al. 2014; Makarikov et al. 2012, 2013, 2015). Archival deposition of georeferenced specimens from inventory remains a basic foundation for characterization of faunal structure (e.g., Hoberg et al. 2009) and is increasingly necessary given the expanding recognition of cryptic diversity across many groups of parasites and hosts (Pérez-Ponce de León and Nadler 2010). The nature of accelerating ecological perturbation, which influences the persistence and distribution of biodiverse systems, emphasizes the immediate need for accurate representations of faunal structure (Brooks et al. 2014; Hoberg et al. 2015).

Among an assemblage of hymenolepidid species, *Hymenolepis folkertsi* Makarikov, Nims, Galbreath and Hoberg 2015, was described based on a limited number of gravid tapeworms in 2 of 20 specimens of the oldfield mouse, *Peromyscus polionotus* (Wagner), from Georgia, USA, in southeastern North America. Comparative morphology provided a capacity to unequivocally distinguish *H. folkertsi* from a limited number of species, with testes disposed in a triangle and an unarmed scolex with a protrusible rostrum-like apparatus, currently known in the North American fauna (Makarikov et al. 2015); e.g., *Hymenolepis pitymi* Yarinsky 1952 in *Microtus pinetorum* (LeConte) (Cricetidae, Arvicolinae) from Tennessee, and *Hymenolepis tualatinensis* Gardner 1985 in *Thomomys bulbivorus* (Richardson) (Geomyidae) from western Oregon (Yarinsky 1952; Gardner 1985). Although clearly differentiated from seven congeneric species in the Nearctic, including *Hymenolepis diminuta* (Rudolphi, 1819), with respect to a suite of structural attributes, the type specimens for *H. folkertsi* were originally fixed in 5 % formalin (Makarikov et al. 2015). Consequently, these specimens were not appropriate for comparisons of molecular sequence data among other species of *Hymenolepis* which could reflect, through integrated approaches, on species identity, higher level relationships, and biogeography (e.g., Haukisalmi et al. 2010).

Coincidental with the description of *H. folkertsi*, ongoing survey and inventory, contributing to broadening museum archives of specimens and tissues documenting rodent helminth faunas across localities in North America, revealed additional specimens of this hymenolepidid; previously unknown specimens were also discovered in privately held collections and among materials archived in the Museum of Southwestern Biology and in the former US National Parasite Collection, now within the US National Museum, Smithsonian. A series of gravid specimens are attributed to *H. folkertsi* from (i) the Upper Peninsula of Michigan, USA [in *Peromyscus maniculatus* (Wagner), *Peromyscus leucopus* (Rafinesque), and *Tamias striatus* (Linnaeus), Sciuridae, *Tamias* Illiger, subgenus *Neotamias* Howell]; (ii) from Pennsylvania, USA (in *P. leucopus*); (iii) from Virginia, USA (in *T. striatus*); (iv) from Connecticut, USA (in *T. striatus*); (v) from south-central Wisconsin, USA [in *T. striatus*, *Ictidomys tridecemlineatus* (Mitchill), *Sciurus carolinensis* Gmelin and *Sciurus niger* Linnaeus], and (vi) from central Idaho, USA (in *Tamias amoenus* Allen) (Fig. 1). Additionally, cestodes in the golden mouse, *Ochrotomys nuttalli* (Harlan) (Neotominae), from adjacent to the type locality for *H. folkertsi* in Georgia were examined from the former US National Parasite Collection. Discovery and collection of these specimens provide the opportunity to complete initial molecular-based comparisons confirming the identity of *H. folkertsi* and to provisionally place this hymenolepidid in a larger phylogenetic context for the genus. Concurrently, we explore evidence for a considerably more extensive host and geographic distribution for *H. folkertsi*, minimally extending across the temperate zone of North America.

**Fig. 1:**
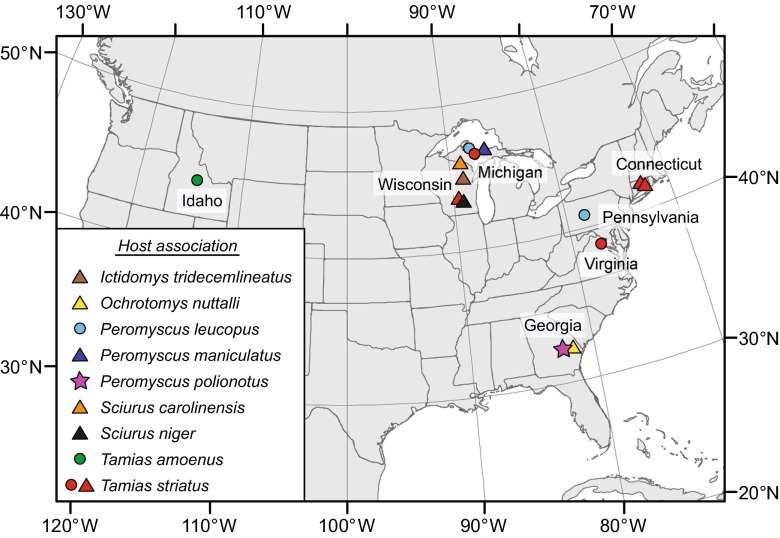
Map of collecting localities for *Hymenolepis folkertsi* specimens. The *star* identifies the type locality for *H. folkertsi. Circles* denote localities from which molecular data were obtained for phylogenetic analyses. *Triangles* indicate localities for which only slide-mounted specimens (no DNA) are available. *Symbol-fill colors* identify the host species represented at each locality (see key). Some localities include multiple collection events from single host species (Alger, Co., MI; Custer Co., ID; Fairfax Co., VA)

## Materials and methods

### Specimens examined and characterized

A series of tapeworm specimens attributed to *H. folkertsi*, as outlined below, was collected from rodent hosts based on field inventory at localities across temperate North America. Tapeworm specimens were collected live, allowed to relax and were heat killed in water, fixed in 10 % formalin or alcohol-formalin-acetic acid (AFA), or preserved in 70 % ethanol and later prepared as permanent vouchers stained in Semichon’s acetic carmine or alum carmine, dehydrated by standard techniques, cleared in xylene, and mounted entirely in Canada balsam. Each specimen (excluding those from Alger County, MI, Tolland County, CT and Fairfax County, VA, in 2008, central WI, and those from GA, all previously fixed in 5 or 10 % formalin or AFA, or already permanently mounted) was subsampled for sequence analysis by the removal of five to six terminal proglottids from the strobila. Subsamples destined for sequencing were subsequently held in individual cryo-vials in 95 % ethanol at ultra-low temperatures, −80 °C, prior to further processing. Cestode specimens, either as permanent slides or in 95 % ethanol frozen storage, are archived in the Parasitology Division of the Museum of Southwestern Biology (MSB), University of New Mexico, Albuquerque, NM; collections of the Harold W. Manter Laboratory (HWML), University of Nebraska; and National Museum of Natural History, Smithsonian Institution (USNM), including the former US National Parasite Collection (USNPC). Databases for host and parasite specimens and associated collection records in part are held in the Arctos database system http://arctos.database.museum. Mammalian taxonomy is consistent with Musser and Carelton (2005) for Cricetidae, Thorington and Hoffmann (2005) for Sciuridae, and Helgen et al. (2009) for ground squirrels.

Hymenolepidid tapeworms examined herein were obtained in specimens of rodent hosts: (i) three cestodes in a *Peromyscus leucopus* from Marquette County in northern MI (Northern Michigan University Museum of Zoology, NMUMZ Catalog No. 530; locality 46.8628° N, 87.7514° W; 22 June 2013 by KEG), under Museum of Southwestern Biology, MSB Catalog No. 23503; (ii) one cestode in a specimen of *T. striatus* from Marquette County in northern MI (NMUMZ No. 695; locality 46.56372° N, 87.41603° W; 15 May 2014 by KEG), under MSB 23504; (iii) two hymenolepidid specimens were obtained in a specimen of *P. maniculatus* from a site in Alger County, MI (University of Michigan Museum of Zoology Catalog No. 169568; locality 46.5894° N, 86.2712° W; 17 July 1991 by SAM) under MSB 23505; other fragmented specimens from two *P. maniculatus* at this site are included under MSB 25310 and MSB 25311. Additional fragmented cestode specimens in *P. maniculatus* were obtained from sites near Pictured Rocks National Lakeshore, Alger County, MI (locality ca. 46.55626° N, 86.29275° W; 2 July and 28 August 1993 by SAM) under MSB 25312 and MSB 25313. (iv) One tapeworm was obtained in a specimen of *P. leucopus* from near Black Moshannon State Park, Centre County, PA (locality 40.87200° N, 78.086105° W; 17 June 2004 by K. Vandegrift to V. Tkach), under MSB 25314. (v) Three fragmented hymenolepidid specimens lacking scolices and two entire cestodes were obtained in separate specimens of *T. striatus* from Annandale, Fairfax County, VA (locality 38.8567616° N, 77.3109676° W; 19 September 2008 and 1 August 2015 by R.P. Eckerlin) under USNM 1418122 and MSB 25315. (vi) One specimen lacking a scolex was collected in *T. striatus* from Mansfield, Tolland County, CT (locality 41.7885° N, 72.2293° W; 9 December 1967 by R.P. Eckerlin) under USNM 1408262. (vii) A single complete cestode in *T. striatus* was collected adjacent to Madison, Dane County, WI (locality ca. 43.0731° N, 89.4012° W; 10 June 1947 by R.L. Rausch) under MSB 24960, originally reported as *H. diminuta* by Rausch and Tiner (1948). (viii) In the upper Midwest based on extensive survey in the late 1940s (Rausch and Tiner 1948), additional specimens originally reported as *H. diminuta* and now attributable to *H. folkertsi* were collected in a single *I. tridecemlineatus* near Shawano, Shawano County, WI (locality ca. 44.77667° N, 88.60194° W; 19 May 1947, by R.L. Rausch; MSB 24966), and in two *S. carolinensis* adjacent to Phelps, Villas County, WI (locality ca. 46.06° N, 89.0908° W; 1 January and 8 July 1948, by R.L. Rausch; MSB 24968, 24971); two incomplete specimens, now considered as *H.* cf. *folkertsi*, had also been collected adjacent to Madison, WI, in 2 *S. niger* (1 October 1946 and 8 February 1947, by R.L. Rausch; MSB 24972, 24974). (ix) Numerous tapeworms, including 11 specimens in *Tamias amoenus*, were collected from sites adjacent to Little Bay Horse Lake, Custer County, ID, in the Salmon-Challis National Forest (locality 16 km SW of Challis, ID, 44.4195° N, 114.3885° W and 44.41485° N, 114.38775° W; 26–27 June 2014, by EPH) with three permanently mounted cestode specimens under MSB 23512, 23513, and 23514. (x) Cestodes were collected in a golden mouse, *O. nuttalli*, on the Charles Harrold Preserve (Nature Conservancy) in Candler County, GA, adjacent to the type locality for *H. folkertsi* (locality 32.418700° N, 82.032670° W; 6 May 2003 by O. Pung and R.P. Eckerlin), with multiple slides including fragments of strobila under USNPC 108204.

### DNA sequencing and genetic analyses

To characterize genetic diversity of specimens identified morphologically as *H. folkertsi* and to quantify their genetic distinctiveness relative to other hymenolepidid species, we collected DNA sequence data from available specimens representing the putative populations in Michigan (three individuals in *P. leucopus*; one individual in *T. striatus*), Pennsylvania (one individual in *P. leucopus*), Virginia (one individual in *Tamias striatus*), and Idaho (three individuals in *T. amoenus*). We sequenced a portion of the mitochondrial NADH dehydrogenase subunit 1 (nad1; ~820 base pairs). Whole genomic DNA was extracted from tissue subsamples (≈3 posterior proglottids) by using Qiagen™ DNeasy Tissue Kits®. We PCR amplified nad1 by using primers nad1f (5′ GGNTATTSTCARTNTCGTAAGGG) and trnNR (5′ TTCYTGAAGTTAACAGCATCA) (Littlewood et al. 2008) in 20 μl volumes, with final reagent concentrations of 1.5 mM Mg^2+^, 0.5 μM primers, 0.4 mM dNTPs, 0.5 U Taq polymerase, and approximately 5 ng/μl template DNA. Standard reaction conditions included a 3-min initial denaturation (94 °C), 30 cycles of 15-s denaturation (94 °C), 30-s annealing (45 °C), 30-s extension (72 °C), and a final 10-min extension (72 °C). All PCR products were sequenced in both directions on ABI 3730 genetic analyzers (Applied Biosystems Inc., Foster City, CA, USA) by using ABI PRISM® BigDyeTM sequencing chemistry. DNA sequences were aligned by eye.

For comparisons to other hymenolepidid diversity, we used additional DNA sequences from GenBank representing *H. diminuta* (GenBank # HM149291), *Arostrilepis beringiensis* (Kontrimavichus and Smirnova, 1991) (KM516216), and *Staphylocystis furcata* (Stieda, 1862) (HM149293). We identified an appropriate model of nucleotide substitution by using the Akaike information criterion to compare 88 alternative models in jModeltest v2.1.5 (Darriba et al. 2012) and used the selected model (HKY+G) to construct a maximum likelihood phylogeny by using Garli v2.0 (Zwickl 2006). We assessed support for relationships with 100 bootstrap replicates. To quantify genetic divergence, we calculated pairwise uncorrected genetic distances and total nucleotide differences between unique haplotypes.

## Results

### Morphological comparisons

Primary diagnostic attributes for a series of specimens in *Peromyscus* spp. and *Tamias* spp. are summarized for comparison to the original description of *H. folkertsi* (Makarikov et al. 2015). Although specimens in *T. striatus* from CT and WI, *Sciurus* spp. from WI, and *O. nuttali* from GA could be identified, these tapeworms were fragmented, incomplete, and not suitable for detailed examination and characterization. As a limited number of high-quality tapeworms were available across disparate geographic sites and host species, characters from representative specimens, or a series at a single locality or in a single host, are presented separately. Each specimen, except those from Alger County, MI, GA, CT, WI, and VA (from 2008), was concurrently sequenced as previously outlined. Measurements are presented in micrometers, unless specified otherwise.

### Specimens in *Peromyscus* spp.

One specimen in *P. leucopus* (Marquette, MI): Scolex 191 wide, with prominent rhynchus; total length of strobila 135 mm, 1.58 mm in maximum width when gravid. Cirrus sac 137–152 (143) long in mature proglottids; 157–166 (159) in post mature proglottids; attaining but not overlapping poral osmoregulatory canals. Cirrus cylindrical. Testes disposed in flat triangle. Ovary 206–230 (214) in maximum width. Gravid proglottids substantially wider than long, 396 in length, 1.58 mm in maximum width; L/W ratio 1:3.98.

Two specimens in *P. maniculatus* (Alger, MI): Scolex 133–182 wide, with prominent rhynchus; total length of strobila not determined in either specimen, 1.89–2.08 mm in maximum width when gravid. Cirrus sac 143–182 (156) in mature proglottids; attaining but not overlapping poral osmogregulatory canals. Cirrus cylindrical. Testes disposed in flat triangle. Ovary 245–264 (251) in maximum width. Embryonic hooks delicate, antero-lateral 16.3, posterolateral 15.3–15.7, medial 16.3–17. Uterus occupying entire proglottid. Gravid proglottid substantially wider than long, 346 in length, 2.08 mm in maximum width; L/W ratio 1:6.01.

One specimen in *P. leucopus* (PA): Scolex 185, with prominent rhynchus and suckers extending beyond lateral margins of scolex, 114–119 × 73–80. Rostellar pouch 121 × 72, not extending beyond posterior margins of suckers. Total length of strobila could not be measured as the specimen is incomplete, 0.968 mm in maximum width at terminal part of strobila (only pregravid proglottids are available, no gravid proglottids were measured). Testes normally situated in triangle with flat angle, 95–110 × 80–96 (103 × 88). Cirrus sac 137–148 (143) in mature proglottids crossing or overlapping poral ventral osmoregulatory canal. Cirrus cylindrical. Ovary 193–232 (214) in maximum width, slightly overlapping testes. Developing uterus occupying entire pregravid proglottid.

### Specimens in *Tamias* spp.

One specimen in *T. striatus* (Marquette, MI): Scolex 157 wide, with prominent rhynchus. Total length of strobila 94 mm, 0.643 mm in maximum width when gravid. Cirrus sac 99–130 (119) in mature proglottids; attaining, slightly overlapping poral osmoregulatory canals. Cirrus cylindrical. Ovary 177–195 (189) in maximum width. Uterus occupying entire proglottid. Gravid proglottids not substantially wider than long, 445 in length, 643 in maximum width; L/W ratio 1:1.44.

Two specimens in *T.* striatus (VA; from 2015): Scolex 167-211, with prominent rhynchus and suckers extending beyond lateral margins of scolex, 114–120 × 82–95 (116 × 89). Rostellar pouch 123-127 × 67-68, not extending beyond posterior margins of suckers. Total length of strobila could not be measured as the specimen is incomplete. Testes normally situated in triangle with flat angle, 115–132 × 107–127 (123 × 114). Cirrus sac in mature proglottids overlapping but not crossing poral ventral osmoregulatory canal. Cirrus cylindrical. Ovary 148–207 (172) in maximum width. Uterus occupying entire proglottid.

Three specimens in *T. amoenus* (ID): Scolex 187–237 wide, with prominent rhynchus; suckers slightly oblong, overlapping lateral margins of scolex, 112–120 (115) long and 86–96 (90) wide. Total length of strobila 110–188.5 mm, 1.108–1.841 mm in maximum width when gravid. Cirrus sac 148–190 (164) in mature proglottids, attaining in two specimens, overlapping poral ventral osmoregulatory canal in one specimen. Cirrus cylindrical. Disposition of testes in shallow triangle. Ovary 151–208 (180) in maximum width. Uterus occupying entire gravid proglottid. Gravid proglottids consistently wider than long, variation evident, L/W ratio in gravid proglottids ranging from 1:3.9 to 4.13 (in one specimen, 1:1.6–2.5).

The specimen in *T. striatus* from Michigan is characterized by a very narrow strobila, and the cirrus sac is notably reduced in length in comparison to the types and original description (Makarikov et al. 2015) and to other conspecific cestodes in *Peromyscus* from adjacent localities in Michigan. Further, in contrast to most other specimens, the cirrus sac overlaps the poral osmoregulatory canals. The L/W ratio in gravid segments is 1:1.44 in this cestode from *T. striatus* but ranges from 1:3.98 to 6.01, and proglottids are substantially wider than long, among other cestodes in *Peromyscus*. Among specimens in *T. amoenus* from Idaho, one also has a relatively narrow strobila, whereas other tapeworms are more typical of the series included in the original description of *H. folkertsi*. This variation may reflect age of particular tapeworms or may be related to development in a species of *Tamias*. Generally, with respect to a series of morphological attributes, these specimens in *Tamias* spp., *Peromyscus* spp., and *O. nuttalli* appear consistent with the original description based on cestodes in *P. polionotus* from Georgia, USA.

### Other specimens in sciuridae

Archival voucher specimens originally reported as *H. diminuta* by Rausch and Tiner (1948) in *T. striatus*, *I. tridecemlineatus*, *S. niger*, and *S. carolinensis* from Wisconsin were redetermined. Consistent with *H. folkertsi*, the scolex (when present) ranged from 160 to 170 in width and a prominent rhynchus was evident; the cirrus pouch length ranged from 119 to 160 and attained and occasionally crossed the ventral osmoregulatory canal. The fully developed uterus filled the proglottid, extending beyond the osmoregulatory canals and contained numerous oval eggs about 55–62 in length.

### Molecular phylogenetics and divergence

**Fig. 2:**
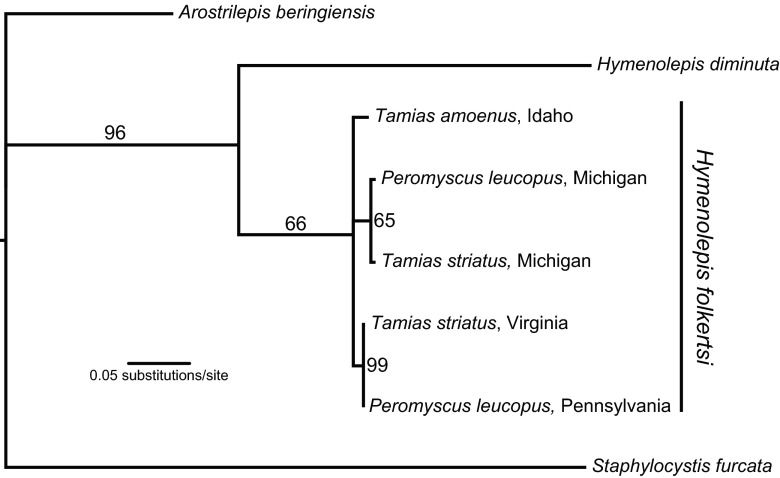
Maximum likelihood phylogeny of *Hymenolepis folkertsi* based on sequences from nad-1 mitochondrial DNA. Within the in-group, branch tips are denoted by host species and geographic locality. Out-groups are denoted by species names. *Numbers adjacent to nodes* denote bootstrap values

DNA sequences obtained from the three tapeworms recovered in a single *P. leucopus* specimen from Michigan were identical, and therefore, we used a single representative sequence for further analyses. Likewise, sequences obtained from the three tapeworms representing different *T. amoenus* specimens from Idaho were also identical and reduced to a single representative for subsequent analyses. Phylogenetic analysis shows that all putative specimens of *H. folkertsi* cluster into a single clade that is most closely related to, and distinct from, *H. diminuta* based on current comparisons (Fig. 2); genetic divergence relative to *H. diminuta* is about 15 %. Within this clade, there is additional geographic structure evident, with distinct haplotypes, respectively, for tapeworms from Michigan, the eastern US (VA + PA), and the western US (ID). Though the current dataset does not resolve relationships among these regional groupings, it is clear that phylogenetic structure is defined by geography rather than associations with sciurid or cricetid hosts. Genetic divergence between *H. folkertsi* specimens (up to 2.7 %) was shallow relative to interspecific divergences (Table 1) and provisionally may be consistent with intraspecific genetic variability estimated for other hymenolepidid tapeworms in rodents (Makarikov et al. 2013). Sequence data for this series of specimens has been deposited in GenBank under the following numbers: GenBank KX782315 (Pennsylvania), KX782314 (Virginia), KX792193-KX792196 (Michigan), and KX792197-KX792199 (Idaho).

**Table 1:** Pairwise uncorrected genetic distances (below diagonal) and total nucleotide differences (above diagonal) in mitochondrial NADH dehydrogenase subunit 1 between putative *Hymenolepis folkertsi* specimens and *Hymenolepis diminuta*

	1.	2.	3.	4.	5.	6.
1. *Hymenolepis diminuta*	–	118	119	116	117	117
2. *Hymenolepis folkertsi* (Pennsylvania, *Peromyscus leucopus*)	0.1535	–	1	19	19	15
3. *Hymenolepis folkertsi* (Virginia, *Tamias striatus*)	0.1550	0.0013	–	19	19	15
4. *Hymenolepis folkertsi* (Michigan, *Peromyscus leucopus*)	0.151	0.0238	0.0241	–	6	22
5. *Hymenolepis folkertsi* (Michigan, *Tamias striatus*)	0.1523	0.0235	0.0238	0.0075	–	22
6. *Hymenolepis folkertsi* (Idaho, *Tamias amoenus*)	0.1522	0.0185	0.0189	0.0276	0.0272	–

The geographic origin and host association for each *H. folkertsi* specimen are listed

### Geographic distribution

Considering these localities and potential rodent hosts, hymenolepidid tapeworms were rare. (i) At the type locality in 2003, *H. folkertsi* occurred in 2 of 20 oldfield mice (10 %) (see Makarikov et al. 2015); adjacent to the type locality, cestodes were collected from 1 of 13 specimens of the golden mouse, *O. nuttalli* (7 %) on the Charles Harrold Preserve (Nature Conservancy) in Candler County, GA, on 6 May 2003. During June–August 2003, 2 golden mice and 20 oldfield mice from two private holdings near Middleground, Bulloch County (respectively, 32.543379° N, 81.832506° W, and 32.561826° N, 81.818846° W) (T.N. Nims, unpublished data) were not infected. (ii) In Virginia, over multiple years of collection, 2 of 22 *T. striatus* (aggregate 9 % over six localities) were found to be infected and only at Annandale, Fairfax County. Parasites were not found in chipmunks examined from Highland County (4), Augusta County (2), Prince William County (1), Rockbridge County (1), and Tazewell County (1) (R.P. Eckerlin, unpublished data). Further, 102 eastern gray squirrels, *S. carolinensis*, examined from sites in Virginia over multiple years were not infected with *H. folkertsi*. (iii) In Pennsylvania, prevalence in *P. leucopus* or other rodents was not determined. (iv) In Connecticut, with multiple years of collection, one specimen of *H. folkertsi* was found in 1 of 19 *T. striatus* from Tolland County (a second lot of tapeworms collected from one chipmunk in 1969 could not be identified), but not in 14 chipmunks from Windham County (aggregate 3 % over both localities); specimens of 62 *S. carolinensis* were not found to be infected (R.P. Eckerlin, unpublished data). Across other widespread localities in the eastern USA, *H. folkertsi* has not been found among other sciurids including 34 eastern fox squirrels *S. niger*, 14 red squirrels *Tamiasciurus hudsonicus* (Erxleben), 3 northern flying squirrels *Glaucomys sabrinus* (Shaw), 21 southern flying squirrels *Glaucomys volans* (Linnaeus), or 8 ground hogs *Marmota monax* (Linnaeus) and among cricetids including 7 southern bog lemmings *Synaptomys cooperi* (Baird), 17 southern rock voles *Microtus chrotorrhinus* (Miller), 8 meadow voles *Microtus pennsylvanicus* (Ord), 3 southern redbacked voles *Myodes gapperi* (Vigors) and 8 white-footed deer mice *P. leucopus* (R.P. Ecklerlin, unpublished data). (v) At localities on the Upper Peninsula of Michigan near Marquette, between 2011 and 2015, specimens attributed to *H. folkertsi* were found in 2 of 27 *T. striatus* (7 %) and one of 44 *P. leucopus* (2 %); specimens of 8 *Tamias minimus* Bachman and 43 *P. maniculatus* were not infected (K.E. Galbreath, unpublished data). (vi) Elsewhere on the Upper Peninsula, from sites in Alger County, *H. folkertsi* was collected in an undetermined number of *P. maniculatus* on 17 July 1991 and in 2 of 47 *P. maniculatus* (4 %) during July–August 1993; seven specimens of *P. maniculatus* from a site at Kingston Plains (46.55608° N, 86.18834° W) on 1 July 1993 were not infected (S.A. Meagher, unpublished data). (vii) In Wisconsin, *H. folkertsi* (reported as *H. diminuta*) was found in 1 of 43 *T. striatus* (2 %), 2 of 94 *S. niger* (2 %), a single *I. tridecemlineatums*, and 2 *S. carolinensis*, although prevalence among these latter host species cannot now be estimated due to the absence of a complete series of specific vouchers and mixed infections involving multiple species of cestodes (see Rausch and Tiner 1948). Further, hymenolepidids were largely absent from this large and extensive sampling of 344 sciurids of 10 species from the upper Midwest. (viii) In Idaho, aside from those in 3 of 12 *T. striatus* (25 %), tapeworms were not found in 3 specimens of *T. hudsonicus*, 8 *Urocitellus columbianus* (Ord), 9 *P. maniculatus*, 1 *Peromyscus truei* (Shufeldt), 4 *Zapus princeps* Allen, 4 *Microtus pennsylvanicus* (Ord), 2 *M. montanus* (Peale), and 2 *Microtus longicaudus* (Merriam) collected adjacent to Little Bay Horse Lake in June 2014. (ix) Hymenolepidids were not found in 11 *P. maniculatus*, 1 *Peromyscus boylii* (Baird), 10 *Z. princeps*, 2 *M. longicaudus*, and 2 *Tamias quadrivittatus* (Say) adjacent to Cabresto Creek, northern New Mexico (5 km N and 13 km E of Questa; ca., 36.733° N, 105.499° W) during inventory on 23–24 June 2014.

## Discussion

### Defining diversity and identity of *H. folkertsi*

Hymenolepidid tapeworms attributed to *H. folkertsi* in cricetid and sciurid hosts during the present study were generally consistent with the type specimens and original description based on a suite of qualitative characters (Makarikov et al. 2015): (1) unarmed scolex with prominent rhynchus; (2) suckers extending beyond lateral margins of the scolex, and the rostellar pouch reaching to the midline but not extending beyond the posterior margins of the suckers; (3) the testes normally situated in triangle with a flat angle; (4) the cirrus sac in mature proglottids attaining but rarely overlapping and usually not crossing the poral ventral osmoregulatory canal; (5) the ovary usually not reaching or strongly overlapping the testes; (6) uterus extending laterally substantially beyond the osmoregulatory system; and (7) distinctly oval eggs of relatively small dimensions, within the range of 55–62 μm in length in this series of specimens. No distinct host or geographic variation in qualitative characters was observed in the present material.

In contrast, there is evident variation in some quantitative attributes among the specimens. For example, the length of the cirrus sac in the type specimens is 138–154 (145) μm, whereas in the vouchers from the present study, it distinctly varies between the smallest dimensions, 99-130 (119) μm in a specimen in *T. striatus* (Marquette, MI), to the largest 148-190 (164) μm in specimens in *T. amoenus* (ID). Furthermore, considerable variation is also observed in width of the strobila; in the type specimens, the width in gravid proglottids is 1.58–1.85 mm (L/W = 1:4.98–6.86), whereas in the specimen in *T. striatus* (Marquette, MI), it is considerably less than half as wide (0.643 mm; L/W = 1:1.44). At the same time, the ovarian width does not vary distinctly among the specimens from different hosts and sites of collection. Interestingly, the measurements from the specimens in *Peromyscus* (i.e., *P. maniculatus* from Alger Co., MI, and *P. leucopus* from Marquette, MI and from PA) are more consistent with the original description than those from *Tamias* spp. at any locality.

Several specimens in *Tamias* and *Peromyscus* are substantially different with respect to dimensions of some organs relative to the type series (Makarikov et al. 2015). All individuals are generally consistent, however, with the type specimens based on qualitative characters. Genetic identity based on comparisons of mitochondrial nad-1 appears compatible with conspecificity of these tapeworms (i.e., 2.7 % divergence or less; Table 1). The absence of sequence data from cestodes in the type host and at the original locality in Georgia, however, suggests that placement of the current series of specimens in *H. folkertsi* may require subsequent revision, and the possibility of unrecognized cryptic species cannot be disregarded.

Preliminary interpretations of this limited dataset suggest that *H. folkertsi* is a geographically widespread hymenolepidid in temperate North America with the potential to occur in sciurid and cricetid rodents (Figs. 1 and 2). Overall prevalence and intensity of infections suggest that the parasite is rare and may have a strongly heterogeneous or patchy geographic distribution in the temperate zone. Intraspecific variation among three apparently disjunct and geographically isolated populations attributed to *H. folkertsi* is indicated by uncorrected genetic distances of 2.7 % (Idaho and Michigan), 2.4 % (Virginia + Pennsylvania and Michigan), and 1.89 % (VA + PA and ID). Regionally, within sites and among sympatric host species, variation does not exceed 0.7 % indicating a pool of tapeworms in circulation among species of chipmunks and deer mice (and probably species of *Sciurus*) within landscapes at respective localities. Consequently, geography rather than host association appears to determine distribution of intraspecific genetic lineages. Host colonization between chipmunks and deer mice within regional sites is indicated (Fig. 2). Tree topology and optimization of hosts on this initial phylogenetic hypothesis would suggest ancestral associations in *Tamias* and independent colonization events to *Peromyscus* with circulation among a broader assemblage of sciurids. Comprehensive sampling of potential hosts and geographic regions, along with more robust phylogenetic resolution, will be critical to evaluating this conclusion. New collections from the type locality in Georgia for *H. folkertsi*, and continuing inventory in temperate zone rodents, are necessary to explore diversity, the directionality and timing of host colonization, and the degree of genetic relationship among these widespread and disparate populations extending to western North America. For example, we found a DNA sequence available on GenBank (HM149295) representing an unidentified species of *Hymenolepis* in an unknown species of pocket gopher (*Thomomys* Wied-Neuwied; Geomyidae) from Wyoming clusters phylogentically with *H. folkertsi* from Idaho (not shown). In the absence of a physical voucher specimen to confirm identity, we chose to exclude this data from our analyses, but it hints at a yet broader host and geographic range for *H. folkertsi* than is currently represented in our samples.

Establishing species limits based on integrated molecular and morphological data among hymenolepidid and related cyclophyllidean tapeworms remains challenging. Genetic divergence revealed across localities for *H. folkertsi* suggests historically isolated populations, consistent with extended evolutionary and biogeographic histories for species of *Peromyscus* and *Tamias* in North America (Kurtén and Anderson 1980). Drivers for parasite and host diversification and faunal assembly are evident through recurring episodes of climatological change, ecological perturbation, and geographic fragmentation over about the last three million years from the late Pliocene through the Quaternary (Dragoo et al. 2006; Hoberg et al. 2012; Makarikov et al. 2015; Sullivan et al. 2014; Galbreath and Hoberg 2015; Bell et al. 2016). Divergence documented among putative populations of *H. folkertsi* may indicate poorly differentiated species or a mosaic of discrete genetic variation across spatial scales reflecting local conditions or assemblages. In comparison, studies of the speciose genus *Arostrilepis*, based on mitochondrial cytochrome *b*, revealed interspecific variation (genetic distances) ranging from 4.5 to 15 % among eight congeners and intraspecific variation between 0.1 and 1.8 % (Makarikov et al. 2013; Galbreath et al. 2013). Pending availability of a larger archive of specimens now attributed to *H. folkertsi* and completion of detailed multi-locus comparisons of diversity in this assemblage, we refrain from proposing the occurrence of additional species-level taxa.

### Host range for *H. folkertsi*

*H. folkertsi* was originally described in *P. polionotus*, an endemic rodent species restricted to southeastern North America, although the actual extent of host and geographic range for this hymenolepidid remains to be completely characterized (Fig. 1). Collections associated with the original description revealed *H. folkertsi* to be a rare parasite in oldfield mice (prevalence in 2 of 20 specimens = 10 % at the type locality at Fifteenmile Creek Preserve; absent in 20 additional oldfield mice at an adjacent site on the Charles Harrold Preserve). Specimens of unarmed hymenolepidids have not been observed otherwise in *P. polionotus*, and the fauna in southern Florida included *Rodentolepis nana* (Siebold, 1852), a ubiquitous rodent tapeworm (Kinsella 1991). The sample sizes examined by Kinsella (1991) are notable, representing188 cricetids of three species including 41 *P. polionotus*, 102 *Podomys floridanus* (Chapman), and 86 *Peromyscus gossypinus* (LeConte) from which unarmed hymenolepidids were not revealed. Two cestodes consistent with *H. folkertsi* were found among 1 of 13 specimens (7 %) of *O. nuttalli*, near the type locality in 2003 (T. Nims, unpublished data); at the present time, endoparasites have apparently been incompletely documented from populations of the golden mouse, and other unarmed hymenolepidids have not been recognized (Forrester 1992; Whitaker and Hamilton 1998). The distribution of *H. folkertsi* among *Tamias* and *Sciurus* in Georgia and eastern North America is also poorly known, although it has been demonstrated among chipmunks in Virginia, Connecticut, Michigan, and Wisconsin based on the current study but appears especially rare among tree squirrels; specimens in CT were originally reported as *Hymenolepis* sp. (Eckerlin 1974). Previously, specimens attributed to *H. diminuta* were collected from 6 of 270 (2 %) gray squirrels at localities in Virginia and West Virginia during a broader geographic survey across the southeastern USA during the 1970s (Davidson 1976); identity cannot be confirmed as vouchers were not retained, and these specimens may have in part represented *H. folkertsi*. In contrast, collections extending over 1988 through 1993, with examination of 119 fox squirrels representing two subspecies, did not reveal either armed or unarmed hymenolepidid tapeworms from localities in Florida (Coyner et al. 1996).

The geographic ranges of *P. polionotus* and *T. striatus* are currently disjunct in the area where *H. folkertsi* was originally discovered. The possibility of historical events of host switching is evident or that a broader but currently unrecognized assemblage of cricetid, sciurid, or geomyid rodents may be involved in persistence at varying spatial scales.

Assuming the correct attribution of the current specimens to *H. folkertsi*, multiple species of cricetids, sciurids, and potentially some geomyids may serve as adequate hosts. Considering only recognized cricetid and sciurid host groups, there are 56 species of *Peromyscus*, the monotypic *O. nuttalli*, 23 species of *Tamias* (*Neotamias*), 2 species of *Ictidomys*, and 10 species of *Sciurus* across North America (Musser and Carelton 2005; Thorington and Hoffmann 2005; Helgen et al. 2009; Sullivan et al. 2014). Minimal synoptic information for parasite diversity has been documented in these assemblages. There is also a lack of comprehensive and contemporary survey data among muroid and other rodents at boreal and temperate latitudes in North America (e.g., Makarikov et al. 2015). Interestingly, conspecific tapeworms were not observed in a sympatric assemblage of muroid (Cricetidae-Neotominae—*Peromyscus* and Arvicolinae—*Microtus* Schrank) and dipodoid (Dipodidae—*Zapus* Coeus) rodents, nor in other sciurids examined at the Little Bay Horse site in Idaho and from northern New Mexico, although at the former locality *H. folkertsi* appears to be well established in chipmunks. It is intriguing that prevalence of these tapeworms in samples of *P. leucopus* (2 %; KEG data from 2011 to 2015) and *P. maniculatus* (4 %; SAM data during July–August 1993) from sites in Michigan, in *P. polionotus* (5 %; TNN aggregate data for 2003, representing the type specimens) and *O. nuttalli* (7 %; TNN data) from Georgia, in *T. striatus* or *S. niger* from Wisconsin (2 %, respectively, based on Rausch and Tiner 1948), and in *T. striatus* from Michigan (7 %; KEG data), Virginia (9 %; RPE data), and Connecticut (3 %; RPE data) appears to be minimal, contrasting with 25 % of chipmunks examined at the Idaho site which were shown to be hosts. Multiple species of *Peromyscus* in sympatry, for example, in northern Michigan representing the *P. leucopus* and *P. maniculatus* species groups (Musser and Carelton 2005), were not shown consistently to be hosts of *H. folkertsi*. Deer mice were not observed to be infected from all geographic localities sampled during the present surveys across what appears to be an otherwise extensive range in temperate North America. Additionally, among other cricetids, various arvicoline rodents may not be among the competent hosts of *H. folkertsi*, as cestodes now attributable to this species were not demonstrated in our minimal collections reported here, nor among 648 voles (multiple species of *Microtus*) examined from the region of the north-central states overlapping geographically with the current study (Rausch and Tiner 1949).

Hymenolepidids, often reported as *Hymenolepis* sp., have only been sporadically documented among species of *Peromyscus* (e.g., summarized in part by Makarikov et al. 2015).

Further, among historical collections of 344 sciurids representing 10 species in the north-central USA during the 1940s, single specimens of *Hymenolepis* sp. [a fragment considered to be similar to *Vampirolepis fraterna* (Stiles, 1906), but not identifiable based on current observation] and *H. folkertsi* (reported as *H. diminuta*) were found in 1 of 43 *T. striatus* (2 %) adjacent to Madison, Wisconsin, but none of 11 *T. minimus* from Minnesota (Rausch and Tiner 1948). Specimens later attributed provisionally to *Hymenolepis citelli* (McLeod 1933) by Voge (1952) [originally reported as *H. diminuta* by Rausch and Tiner (1948)] were collected among 13 of 80 13-lined ground squirrels *I. tridecemlineatus* in this Midwestern sample. Also, during this extensive survey, 2 of 94 *S. niger* and 1 of 10 Franklin’s ground squirrels *Policitellus franklinii* (Sabine) were revealed to be hosts for *H. diminuta*, whereas an undetermined *Hymenolepis* sp. was reported based on unpublished records in a series of tree squirrels from Ohio by another collector (1 of 16 *S. niger*, 4 of 72 *S. carolinensis*) (Rausch and Tiner 1948).

An incomplete series of voucher specimens representing material collected directly and examined by Rausch and Tiner (1948) are currently archived in the Rausch Helminthological Collections held by the MSB. The identity of representative specimens as *H. citelli* in this series of sciurids (excluding *T. striatus*) was in part confirmed in *I. tridecemlineatus*, but not in species of *Sciurus* based on examination of a limited number of vouchers during the present study. Among two host specimens of 13-lined ground squirrels, single cestodes were redetermined as *H. folkertsi* (MSB 24966) or provisionally confirmed as *H.* cf. *citelli* (MSB 24967). Among two *S. carolinensis*, three cestode specimens are now referable to *H. folkertsi* (MSB 24968, 24971), whereas among two *S. niger*, two hymenolepidids (MSB 24972, 24974) are now provisionally referred to this species. Additionally, we examined vouchers (MSB 23517, 23518, 23519, and 23520) attributed to *H. diminuta* in rice rats *Oryzomys palustris* (Harlan), where 34 of 178 (19 %) of these sigmodontine cricetids were found to be hosts in a northern Florida marsh (Kinsella 1988). These specimens, here considered to represent a currently undescribed species of *Hymenolepis*, are excluded from either *H. folkertsi* or *H. diminuta* based on attributes of the scolex, an extremely long-neck region (2.4 mm), shape of weakly craspidote proglottids, and dimensions of the cirrus sac and cirrus, along with the shape and diameter of the eggs. Specimens of *H. folkertsi* can be immediately separated from *H. citelli* and *H. diminuta* based on the structure of the scolex (miniscule dimensions and rhynchus) and other attributes including shape and dimensions of the relatively small eggs (McLeod 1933; Voge 1952; Makarikov et al. 2015), indicating that multiple species of *Hymenolepis* are in circulation across a broad assemblage of rodents in the temperate zone. Among these, *H. folkertsi* is now regarded to have an extensive but patchy geographic distribution in association with some cricetids (neotomines including species of *Peromyscus* and *O. nuttalli*) and sciurids (species of *Tamias*, *Ictidomys*, and *Sciurus*) at disparate sites. Although considered to have been resolved, the nature of the independent status of *H. diminuta* and *H. citelli* requires exploration in the context of DNA sequence data from new series of collections.

Additional unsubstantiated records of hymenolepidids based on recovery of cestode eggs in fecal samples from *T. striatus* have also been reported. Across sites in Pennsylvania, separate studies documented prevalence of hymenolepidids among eastern chipmunks with 11 % of 163 (reported as *H. diminuta*) and 2.6 % of 238 (reported as *Hymenolepis* sp.) recognized as hosts (Mahan and Itle 2006; Grear 2011). Although these infections may represent *H. folkertsi*, strobilate adult vouchers were not collected, identified, or archived, and species determination cannot now be confirmed.

Faunal assembly and diversification among rodent tapeworm faunas is complex, reflected in varying temporal and spatial factors that influence biogeography, host range, and patterns of apparent specificity. For example, among species of *Arostrilepis*, primarily among arvicolines, specificity is often manifested at the level of host genus with discrete taxa partitioned among species of *Myodes* Pallas, *Microtus*, and other voles and lemmings (Makarikov et al. 2011, 2013). A role for host colonization, from arvicoline sources, is also evident with species distributed among geomyid, heteromyid, and neotomine rodents (Makarikov et al. 2012). Host switching is apparent in different temporal settings, initially in the origin and later diversification of *Arostrilepis* (among arvicolines) and secondarily in contemporary assemblages involving voles and occasionally sciurids in the Nearctic and Palearctic (Haukisalmi et al. 2010; Makarikov et al. 2013). Sciurids, however, are considered to be incidental hosts for species of *Arostrilepis*, whereas speciation appears to have been driven by associations with arvicolines. In the Palearctic, *Arostrilepis macrocirrosa* Makarikov, Gulyaev et Kontrimavichus, 2011 and *Arostrilepis tenuicirrosa* Makarikov, Gulyaev et Kontrimavichus, 2011, which are considered as specific parasites of red-backed voles (*Myodes*), very sporadically occur in Eurasian red squirrels (*Sciurus vulgaris* Linnaeus) (e.g., Galbreath et al. 2013). Further, in the Nearctic, *A. macrocirrosa* has been observed among red squirrels, *T. hudsonicus*, in areas of sympatry with *Myodes rutilus* (Pallas) (Makarikov et al. 2013). These patterns of diversification and assembly may contrast with the history for *H. folkertsi*, where both cricetids and sciurids represent competent hosts, either in sympatry or in relative isolation, for a cestode that may describe a heterogeneous distribution at local to regional scales.

Shared habitat, ecological similarity, and local guild dynamics related to common food resources for some rodents explain transmission of conspecific hymenolepidids between or among phylogenetically distant hosts. Essentially, successful colonization reflects converging factors for opportunity and capacity, and increasingly, the interactions among recurrent climatological and environmental perturbation, geographic expansion, ecological fitting, and oscillations in host range are regarded as critical inter-related processes (e.g., Agosta et al. 2010; Araujo et al. 2015; Galbreath and Hoberg 2015; Hoberg and Zarlenga 2016). Previously, Dogiel (1962) and Kontrimavichus (1969) noted that ecological similarity of hosts and host switching by parasites (a term they established as “hostal radiation” when involved with parasite diversification) were significant determinants of diversity and helminth community structure. Subsequently, a pervasive role for ecological perturbation, faunal mixing, and host/geographic colonization has been recognized in the process of parasite diversification and as a driver for mosaic faunal assembly and structure in evolutionary and ecological time (e.g., Hoberg and Brooks 2008, 2015; Galbreath and Hoberg 2015; Bell et al. 2016).

Knowledge of parasite diversity is a cornerstone in recognizing and documenting disturbance and faunal perturbation at all biogeographic scales from landscapes to regions. Renewed attention to comprehensive survey and inventory to establish the structure of biodiverse faunas is essential in identifying the outcomes of accelerating change linked to climate warming and anthropogenic forcing (e.g., Brooks et al. 2014). The current study consolidates a range of new and previously unavailable data for faunal structure among rodents and helminths while contributing to developing baselines, either as direct indicators or as proxies, against which environmental change and perturbation may be assessed. Following centuries of parasitological investigation, and the ubiquitous geographic distribution of a diverse assemblage of small mammals, it is somewhat remarkable that a substantial lacuna in knowledge remains apparent with respect to rodent helminth faunas across the temperate zone on the North American continent.
